# Ototoxicity beyond platinum-based derivatives

**DOI:** 10.1007/s00280-026-04944-3

**Published:** 2026-09-09

**Authors:** Cecília Vieira Peruch, Marcia Salgado Machado, Vera Beatris Martins, Felipe de Oliveira Goulart, Monalise Costa Batista Berbert, Eliane Dallegrave

**Affiliations:** 1 Graduate Program in Pathology, Federal University of Health Sciences of Porto Alegre (UFCSPA), Porto Alegre, Rio Grande do Sul Brazil; 2 Santa Casa de Porto Alegre, Porto Alegre, Rio Grande do Sul Brazil; 3 Department of Speech Therapy, Universidade Federal de Ciências da Saúde de Porto Alegre, Porto Alegre, Brasil; 4 Department of Pharmacosciences, Graduate Program in Pathology, Federal University of Health Sciences of Porto Alegre (UFCSPA), St. Sarmento Leite, 287, Porto Alegre, 90050-170 Rio Grande do Sul Brazil; 5 Graduate Program in Rehabilitation Sciences,, Federal University of Health Sciences of Porto Alegre (UFCSPA), Porto Alegre, Rio Grande do Sul Brazil; 6 Department of Speech Therapy, Federal University of Minas Gerais (UFMG), Minas Gerais, Brazil

## Abstract

**Purpose:**

Ototoxicity caused by chemotherapy is a growing problem. Literature highlights the effects of platinum compounds. However, other chemotherapy drugs also have demonstrated audiological changes. The objective of this study was to compare pre- and post-treatment distortion product otoacoustic emissions (DPOAEs) in adult patients undergoing different chemotherapy regimens.

**Methods:**

Cohort study comprising 174 individuals receiving chemotherapy (CT) between April 2022 and December 2023. DPOAE were evaluated in all patients at 2 kHz, 4 kHz, 6 kHz, 8 kHz, 10 kHz, and 12 kHz, before initiating chemotherapy and after completing the treatment. For differential analysis, the protocols were grouped into CT with platinum-based agents and other protocols.

**Results:**

A reduction in response in at least one frequency of the DPOAE test was observed in 79% of the patients. 81% of those who received CT without platinum derivatives exhibited alterations in post-treatment examinations, whereas in the group with platinum-based compounds, alterations were observed in 79% of the subjects. Different percentages of response deterioration were observed across frequencies: 27.6% at 2 kHz, 24.6% at 4 kHz, 41.2% at 6 kHz, 58.8% at 8 kHz, 57.4% at 10 kHz, and 72.4% at 12 kHz. The ototoxic effect of chemotherapy protocols, with and without platinum, was more pronounced at higher frequencies, with no statistically significant difference between the groups.

**Conclusion:**

Chemotherapy has shown signs of ototoxicity through changes in DPOAEs in the sample studied. The ototoxic effects were similar between drug protocols with and without platinum, highlighting the need for audiological monitoring in all chemotherapy patients.

## Introduction

The ototoxicity of Cisplatin and Carboplatin has been extensively studied and well-documented, making it a significant concern during cancer treatment [1]. However, other chemotherapy drugs should also be considered. A recent review [2] identified only six chemotherapeutic agents as ototoxic: Cisplatin, Carboplatin, Vinblastine, Vincristine, Dasatinib, Isotretinoin, and Tretinoin. Nonetheless, additional drugs have been reported in studies and case reports for their harmful effects on hearing. Research on Doxorubicin [3], Docetaxel [4], Paclitaxel [5, 6], Oxaliplatin [7, 8], and FEC [9] (Fluorouracil, Epirubicin, and Cyclophosphamide) has raised concerns after showing evidence of ototoxicity.

For auditory monitoring, Distortion Product Otoacoustic Emissions (DPOAE) have proven to be more effective in detecting ototoxicity, as they cover a wide range of frequencies, including high frequencies, which are the first to be affected by the toxicity of antineoplastic drugs [10]. Additionally, using a non-behavioral method such as DPOAE is recommended due to the fragile health conditions of patients, who may be unable to provide reliable responses in behavioral tests or even move to an audiometric booth. DPOAE can also be used to predict auditory impairment at an earlier stage [11].

Currently, studies on this topic focus primarily on specific drugs, especially platinum-based derivatives. Reports and case series describe auditory changes caused by other chemotherapeutic agents, though with less representative data. In Brazil, in particular, the literature lacks broader studies on ototoxicity related to different antineoplastic protocols in the population, using auditory monitoring methodologies.

Therefore, this study aimed to analyze and compare the ototoxic effects of different chemotherapy treatments on patients’ hearing in the chemotherapy unit of a large hospital in southern Brazil. Additionally, the results, along with a concise and organized compilation of informations, aim to assist healthcare professionals in their clinical decision-making.

### Methods

This study was approved by the Research Ethics Committee of ISCMPA/UFCSPA (Approvals No. 5.117.189/5.284.891) and was registered in Brazil’s National System for Research Registration Involving Human Subjects (Plataforma Brasil – CAAE: 52572021.2.0000.5335).

Prospective cohort study comprising 174 individuals who underwent chemotherapy treatment between April 2022 and December 2023. The inclusion criteria required participants to be 18 years or older, be patients in the oncology service, and have no prior chemotherapy treatment. The exclusion criteria included having a diagnosis of any neoplastic condition affecting the ear or its adjacent structures. Sociodemographic information, medical history, and treatment details were collected directly from electronic medical records, and treatment protocols followed established guidelines for each cancer type and the specific needs of the patients without research group interference. All participants received detailed explanations about the study, the data collected, and their involvement, providing their informed consent by signing the Free and Informed Consent Form.

A visual inspection of the external auditory canal (EAC) was performed using the “Omni 3000 Xenon” otoscope to check for conditions that could restrain the auditory assessment. If any abnormalities were detected during the EAC inspection, the patient was referred for evaluation by a specialist and was excluded from the study. From an initial sample of 300 patients, 63 were excluded due to abnormalities detected in the EAC inspection, resulting in a final pre-chemotherapy (pre-QT) sample of 227 individuals. The auditory tests were conducted with the patient seated on a stretcher or chair in the chemotherapy unit’s consultation or treatment room. Although these rooms did not have acoustic isolation, the environment was quiet, and background noise did not interfere with testing, allowing accurate assessments.

DPOAE testing was conducted using the Otoread Clinical^®^ device (Interacoustics^®^), which captures responses through a miniature microphone placed in the external auditory canal, sealed with a latex ear tip, following calibration standards set by the Federal Council of Speech-Language Pathology and Audiology [12] (Conselho Federal de Fonoaudiologia). DPOAE responses were measured at frequencies of 2, 4, 6, 8, 10, and 12 kHz in both ears, using F1 and F2 tones at stimulus levels of 65 and 55 dB. Signal-to-noise ratio (SNR) values were recorded for each ear and frequency, maintaining numerical integrity. The results were printed directly from the device, digitized, and stored as physical copies.

All assessments were conducted by the lead researcher, who had seven years of experience in audiology and underwent formal training before conducting the study.

After being entered into a data spreadsheet, the DPOAE SNR values for each frequency were analyzed in two ways: first, by identifying the presence or absence of a response in the tested frequencies, considering a response as present if the SNR value was ≥ 6 dB above the noise floor [13, 14], and second, by numerically comparing the exact SNR amplitude values.

The evaluation protocol was conducted before the administration of chemotherapy drugs (pre-QT), during the patient’s first scheduled chemotherapy session. The second assessment was performed between 30 and 90 days after the completion of the treatment protocol (post-QT), during the patient’s follow-up medical consultation.

A total of 29 individuals passed away before the final data collection, and 21 withdrew from the study due to non-attendance or deteriorating health conditions, resulting in a final sample of 174 patients.

Initially, the entire sample was analyzed together. However, given the high ototoxicity of platinum-based compounds, the sample was later divided into two groups for comparative analysis: patients treated with platinum-based chemotherapy protocols (79 individuals) and patients treated with non-platinum-based protocols (95 individuals).

The platinum-based chemotherapy group included the following protocols: BEP (Etoposide, Cisplatin, and Bleomycin), Carboplatin, Carbotaxol (Paclitaxel and Carboplatin), Cisplatin, DHAP (Cisplatin and Cytarabine), EP (Etoposide and Cisplatin), Folfox (Oxaliplatin and Fluorouracil), and Xelox (Oxaliplatin and Capecitabine).

The non-platinum-based chemotherapy group included the protocols ABVD (Doxorubicin, Bleomycin, Vinblastine, and Dacarbazine), AC + T (Doxorubicin, Cyclophosphamide, and Trastuzumab), Capecitabine, CHOEP (Doxorubicin, Vincristine, Cyclophosphamide, and Etoposide), CVP (Cyclophosphamide and Vincristine), Docetaxel, Doxorubicin, Folfiri (Fluorouracil and Irinotecan), Gemcitabine, Paclitaxel (Paclitaxel and Trastuzumab), R-CHOP (Doxorubicin, Vincristine, Cyclophosphamide, and Rituximab), and R-CVP (Rituximab, Cyclophosphamide, and Vincristine).

For statistical analysis of the quantitative variables in response to the exam, the data distribution for each frequency was subjected to the Shapiro-Wilk normality test. It was observed that all quantitative response variables to the exam rejected the normality hypothesis, so the data were summarized and presented as median and interquartile range (Q1 – Q3). The Wilcoxon test, a non-parametric alternative to the paired t-test, was used to compare the values between before (pre-CT) and after treatment (post-CT).

For the analysis of categorical variables, the data were summarized and presented as absolute frequency and proportion. The quantitative response variables to the exam were categorized according to the clinical criterion mentioned. To compare statistics between before and after treatment, the paired McNemar test was used. The quantitative variables used were the SR responses of the EOAPD in their numeric entirety in dB per frequency. All statistical analyses used a 95% confidence level and were performed using R software version 4.3.0.

## Results

A total of 174 patients were analyzed. Table 1 presents the characterization of the sample with the main characteristics relevant to oncological treatment.

**Table 1:** Sample Characterization

	*N* = 174
**Age**	56 (14); 59 [48–66]
**Age Range**	
From 18 to 49 years	49 (28%)
From 50 to 59 years	40 (23%)
From 60 to 69 years	59 (34%)
Over 70 years	26 (15%)
**Sex**	
Female	111 (64%)
Male	63 (36%)
**Ethnicity**	
White	131 (75%)
Brown	28 (16%)
Black	12 (6.9%)
Yellow	2 (1.1%)
Indigenous	1 (0.6%)
**Diabetes**	
No	142 (82%)
Yes	32 (18%)
**Systemic arterial hypertension**	
No	96 (55%)
Yes	78 (45%)
**Smoking**	
No	87 (50%)
Yes	87 (50%)
**Alcoholism**	
No	145 (83%)
Yes	29 (17%)
**Cancer Topography**	
* Breast*	48 (28%)
* Lung*	37 (21%)
* Gynecological*	20 (11%)
* Colorectal*	18 (10%)
* Lymphoma*	16 (9.2%)
* Upper gastrointestinal*	15 (8.6%)
* Urological*	9 (5.2%)
* Head and neck*	8 (4.6%)
* Melanoma*	2 (1.1%)
* Sarcoma*	1 (0.6%)
**Concomitant radiotherapy with chemotherapy**	
No	126 (72%)
Yes	48 (28%)
**Duration of chemotherapy (days)**	99 (64); 86 [44–147]
**Duration of chemotherapy**	
Up to 30 days	22 (13%)
31 to 60 days	29 (17%)
61 to 90 days	37 (21%)
91 to 120 days	26 (15%)
121 to 150 days	29 (17%)
More than 150 days	31 (18%)
**Platinum-based derivatives protocol**	
No	95 (55%)
Yes	79 (45%)

Mean (Standard Deviation); Median [25%-75%]; n (%)

The analysis of DPOAE results was conducted qualitatively by comparing the pre-CT and post-CT results of each subject. A response was considered present when SNR was ≥ 6 dB and absent when < 6 dB. The characteristics were organized by frequency in kHz and ear, with the left ear (LE) and right ear (RE) being analyzed separately. Given that absent responses in the pre-CT would not change state, the qualitative reduction was analyzed only in the present responses (≥ 6 dB). The results shown in Table 2 demonstrated a significant reduction in all tested frequencies.

**Table 2:** DPOAE comparison between pre-CT and post-CT in subjects with response presence at pre-treatment evaluation

Caracteristics	DPOAE pre-CT	DPOAE post-CT		
	present	present	absent	*p*-value
**LE_2kHz**	112 (100%)	90 (80%)	22 (20%)	**< 0**,**001**
**LE_4kHz**	101 (100%)	80 (79%)	21 (21%)	**< 0**,**001**
**LE_6kHz**	83 (100%)	56 (67%)	27 (33%)	**< 0**,**001**
**LE_8kHz**	71 (100%)	38 (54%)	33 (46%)	**< 0**,**001**
**LE_10kHz**	67 (100%)	38 (57%)	29 (43%)	**< 0**,**001**
**LE_12kHz**	38 (100%)	13 (34%)	25 (66%)	**< 0**,**001**
**RE_2kHz**	116 (100%)	94 (81%)	22 (19%)	**< 0**,**001**
**RE _4kHz**	121 (100%)	102 (84%)	19 (16%)	**< 0**,**001**
**RE _6kHz**	80 (100%)	61 (76%)	19 (24%)	**< 0**,**001**
**RE _8kHz**	75 (100%)	38 (51%)	37 (49%)	**< 0**,**001**
**RE _10kHz**	67 (100%)	36 (54%)	31 (46%)	**< 0**,**001**
**RE _12kHz**	37 (100%)	15 (41%)	22 (59%)	**< 0**,**001**

n (%) - McNemar test with continuity correction

A concise summary of the proportion of reduction by frequency, grouping the responses for the left ear (LE) and right ear (RE), and analyzing the loss of responses unilaterally or bilaterally, is presented in Table 3.

**Table 3:** Overall response proportion of DPOAE by frequency in relation to laterality

Frequency	Presence of unilateral or bilateral DPOAE responses	Unilateral DPOAE response loss	Bilateral DPOAE response loss
2 kHz	134 (100%)	37 (27,6%)	7 (5,2%)
4 kHz	130 (100%)	32 (24,6%)	8 (6,2%)
6 kHz	97 (100%)	40 (41,2%)	6 (6,2%)
8 kHz	102 (100%)	60 (58,8%)	10 (9,8%)
10 kHz	94 (100%)	54 (57,4%)	6 (6,4%)
12 kHz	58 (100%)	42 (72,4%)	5 (8,6%)

n (%)

Considering the already proven ototoxicity of platinum-based compounds, the sample was divided into two groups. Group 1 consisted of patients treated with chemotherapy protocols containing platinum-based derivatives, while Group 2 included the remaining protocols. As in the previous analysis, the quantitative reduction analysis considered only the responses present in the pre-treatment (≥ 6). As shown in Table 4, both groups exhibited a reduction in responses across all frequencies (p-value < 0.05, McNemar).

**Table 4:** Comparison between pre- and post-DPOAE stratified by platinum and non-platinum groups, in subjects with response presence at pre-treatment evaluation

	Group 1 – With Platinum-based CT					Group 2 - Without Platinum-based CT				
	DPOAE pre-CT	DPOAE post-CT			DPOAE pre-CT		DPOAE post-CT			
Caracteristics	present	present	absent	*p*-value	present		present	absent	*p*-value	
**LE_2kHz**	52 (100%)	39 (75%)	13 (25%)	**0**,**001**	60 (100%)		51 (85%)	09 (15%)	**0**,**008**	
**LE_4kHz**	41 (100%)	31 (76%)	10 (24%)	**0**,**004**	60 (100%)		49 (82%)	11 (18%)	**0**,**003**	
**LE_6kHz**	31 (100%)	22 (71%)	09 (29%)	**0**,**008**	52 (100%)		34 (65%)	18 (35%)	**<0**,**001**	
**LE_8kHz**	22 (100%)	13 (59%)	09 (41%)	**0**,**008**	49 (100%)		25 (51%)	24 (49%)	**<0**,**001**	
**LE_10kHz**	26 (100%)	12 (46%)	14 (54%)	**0**,**001**	41 (100%)		26 (63%)	15 (37%)	**<0**,**001**	
**LE_12kHz**	15 (100%)	03 (20%)	12 (80%)	**0**,**001**	23 (100%)		10 (43%)	13 (57%)	**0**,**001**	
**RE_2kHz**	56 (100%)	41 (73%)	15 (27%)	**<0**,**001**	60 (100%)		53 (88%)	07 (12%)	**0**,**023**	
**RE_4kHz**	51 (100%)	42 (82%)	09 (18%)	**0**,**008**	70 (100%)		60 (86%)	10 (14%)	**0**,**004**	
**RE_6kHz**	33 (100%)	22 (67%)	11 (33%)	**0**,**003**	47 (100%)		39 (83%)	08 (17%)	**0**,**013**	
**RE_8kHz**	29 (100%)	15 (52%)	14 (48%)	**0**,**001**	46 (100%)		23 (50%)	23 (50%)	**<0**,**001**	
**RE_10kHz**	28 (100%)	12 (43%)	16 (57%)	**<0**,**001**	39 (100%)		24 (62%)	15 (38%)	**<0**,**001**	
**RE_12kHz**	16 (100%)	05 (31%)	11 (69%)	**0**,**003**	21 (100%)		10 (48%)	11 (52%)	**0**,**003**	

n (%) - McNemar test with continuity correction

**Fig. 1:**
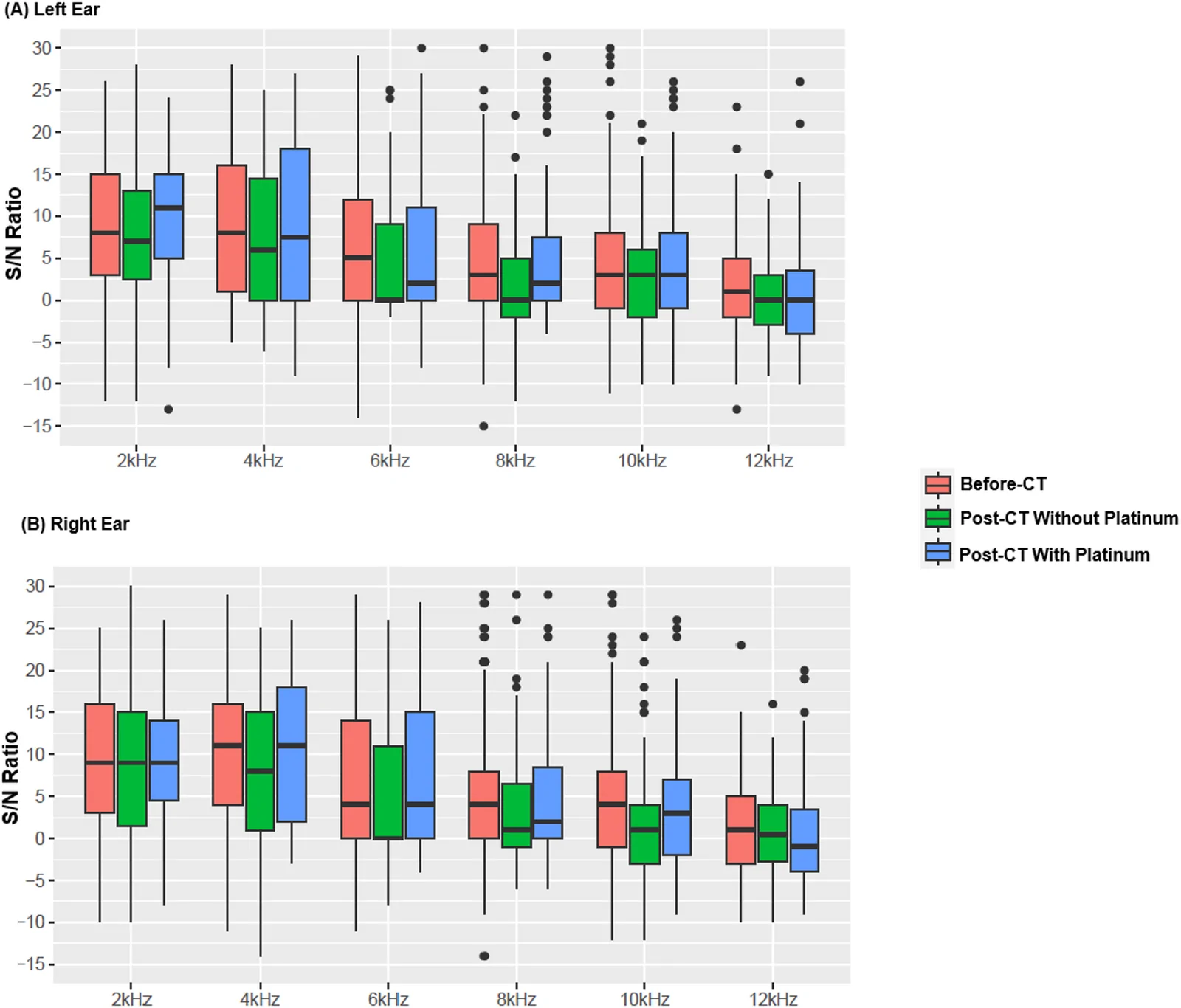
Comparison of DPOAE pre-CT and post-CT chemotherapy in the left ear (**A**) and right ear (**B**)

Figure 1 provides a better visualization of the difference in general DPOAE responses between pre-treatment and post-treatment, stratified by groups. These data are presented for both the left ear (A) and the right ear (B).

Considering the lack of consensus in the literature for ototoxicity criteria, we conducted an analysis based on the criterion of a reduction greater than 6 dB in the SNR, as used in various studies and based on Beattie’s [15] research on the reliability of ototoxic change. Table 5 presents the percentage of patients with a reduction in all individuals, then the values per group, with platinum-based drugs and without platinum-based drugs, along with statistical analysis comparing the results between groups.

**Table 5:** Comparison of the proportion of >6dB worsening between platinum and non-platinum-based groups post-chemotherapy

Frequency	Overall	Platinum-based Derivatives		
	*N* = 174	YES, *N* = 79	NO, *N* = 95	*p*-value
**2 kHz**	57 (33%)	33 (42%)	24 (25%)	**0**,**021**
**4 kHz**	49 (28%)	19 (24%)	30 (32%)	0,272
**6 kHz**	44 (25%)	20 (25%)	24 (25%)	0,994
**8 kHz**	66 (38%)	29 (37%)	37 (39%)	0,762
**10 kHz**	69 (40%)	32 (41%)	37 (39%)	0,834
**12 kHz**	73 (42%)	30 (38%)	43 (45%)	0,332
**At least 1 episode of >6dB reduction**	157 (90%)	69 (87%)	88 (93%)	0,242

n (%) - Chi-square test of independence

## Discussion

To our knowledge, this is the first study to perform broad-spectrum audiological monitoring of adults undergoing chemotherapy across a wide range of treatment protocols. Because this was designed as a screening study including heterogeneous chemotherapy regimens, analyses according to individual chemotherapeutic agents, cumulative doses, and number of treatment cycles were beyond the scope of the present investigation. The primary objective was to determine whether evidence of ototoxicity could also be identified in patients receiving chemotherapy regimens without platinum derivatives.

In general, the most affected frequencies were 6 kHz, 8 kHz, 10 kHz, and 12 kHz, consistent with the existing literature [16, 17]. Significant changes were found across all frequencies, starting with a 20% (22) reduction at 2 kHz and reaching up to 66% (25) at 12 kHz. Demonstrating that higher frequencies exhibited a greater reduction. The same pattern was observed in the quantitative analysis, where the reduction in the S/N was statistically significant across all frequencies. These findings are consistent with the study by Biro et al. (2006) [18], which also observed changes in DPOAEs at mid frequencies (1 kHz, 2 kHz, 3 kHz and 4 kHz) among individuals who received Cisplatin.

Currently, an increasing number of studies highlighted the relevance of high frequencies for speech recognition [19, 20]. One study involving middle-aged adults with normal hearing found significant correlations between lower hearing thresholds in high frequencies and lower scores in self-perception and speech intelligibility assessments [21]. Ultra-high frequencies also play a crucial role in monitoring, as they can predict future changes and self-reported difficulties [11, 22].

Cisplatin and carboplatin are the chemotherapy drugs most linked to hearing loss. A review [2] analyzed over 2,000 articles published in the past decade on chemotherapy-induced ototoxicity, finding that platinum-based compounds are the most frequently reported drugs in the literature, making up two-thirds of the studies on ototoxicity in cancer treatments. Oxaliplatin was not considered ototoxic in a clinical trial with only 18 patients [23]. However, case reports have indicated that the drug may cause hearing loss [7, 24, 25].

Given that the high toxicity of platinum-based compounds could be affecting the responses observed in this study, we divided the sample into two groups. In this analysis, 76% of individuals undergoing chemotherapy without platinum-derived compounds exhibited significant changes in DPOAEs at least in one frequency. This finding demonstrates similar results with a study in which, when comparing treatments between Taxanes and Platinum, the authors were surprised to find significant auditory changes in the group receiving Taxanes alone [6]. When comparing the groups in the present study, the levels of ototoxicity were similar, with no statistically significant differences between them.

Studies on Vincristine and Vinblastine have raised concerns regarding the potential effects of these drugs on the auditory system, although the changes observed were less significant compared to those of platinum-based compounds [26–28]. Among other chemotherapy drugs not commonly associated with ototoxicity as a side effect, Doxorubicin has shown ototoxic effects in animal studies, but there is a lack of supporting literature [2]. Another study involving the FEC protocol (Fluorouracil, Epirubicin, and Cyclophosphamide) reported atypical hearing loss in 9 out of 16 women treated (64%) [9].

In the Taxane class, the ototoxicity of Docetaxel is reported in a case study with two patients [4]. Case reports of auditory toxicity have also been found with the use of Paclitaxel [5, 29]. Another study compared the ototoxic effects in patients treated with cisplatin, taxanes, and a combined therapy of both, finding ototoxic outcomes in all groups [6]. These results are consistent with those of the present study, showing little to no difference in the ototoxicity observed between the two groups.

The present study also found differences in the responses between the left and right ears, which contrast with other studies showing bilateral and symmetric auditory changes due to ototoxicity [32, 33]. Given that ototoxicity is considered a systemic toxicity, many studies group ear responses by frequency [30, 31]. However, some studies have analyzed the ears separately and found different responses [10, 34, 35]. The study by Budai et al. (2020) [34] demonstrated the presence of genes that could be determinants for cisplatin-induced ototoxicity, classifying it into unilateral or bilateral involvement. This suggests that the presence of different changes between ears is possible in some cases. Another publication [35] investigated the hearing of 200 children undergoing chemotherapy, showing that chemotherapy involving cisplatin caused hearing loss in 41.9% of right ears and 47.3% of left ears.

Due to the high average age, many of the individuals tested showed absent OAEs at certain frequencies in the pre-treatment exam, even before exposure. Therefore, the responses were analyzed by frequency and ear, allowing for a detailed and independent transition overview. Given this, analyses were performed only with the responses present, a practice also adopted in other studies that aim for a more accurate assessment of changes [6].

According to INCA [36], most cancer cases occur after the age of 50. Around 60% of cancer patients undergoing treatment are considered elderly, being over 50 years old. Thus, it is crucial to consider that patients with pre-existing hearing impairments may experience further deterioration following treatment. One study reported that older patients showed a significant incidence of pre-existing audiometric changes, with no significant difference in the incidence of post-chemotherapy audiometric changes when compared to the group without previous impairments [37]. However, in our study, despite the similar results, the importance of choosing the right test for auditory monitoring is emphasized, as OAEs are predictive of cochlear function changes that occur before the alterations detected in conventional audiometry [38–40].

These data highlight the need for attention to individuals over 50 years old undergoing chemotherapy, as more factors are associated with the threat to auditory health. These patients are more vulnerable to ototoxic medications due to age-related renal function decline [41, 42], and other risk factors such as chronic diseases [43]. Neurotoxicity may also be linked to poorer outcomes and quality of life in cancer survivors [43, 44]. The strong impact of hearing loss and the increased risk for depression and dementia, which are directly related to quality of life, are well documented [45, 46].

By measuring DPOAE, these patients can be identified before significant auditory functional impairments are detected by other audiological tests. Studies have shown that DPOAE results are predictors of future auditory changes, often being the first to display the effects of ototoxicity [19, 21, 47]. Thus, they are recommended as markers for changes that may not yet be present in other audiological assessments [10].

Our findings suggest that auditory monitoring should be applied to all patients undergoing chemotherapy. Further research within the oncology population, with different chemotherapy agents, is essential, as there is an urgent need to mitigate the impacts of ototoxicity in cancer patients. Early detection of chemotherapy-induced hearing loss is therefore crucial for patients undergoing chemotherapy. OAEs are a sensitive and efficient tool for early detection of ototoxicity, as they do not require behavioral responses. Addressing these patients’ auditory health can be an integral part of the therapeutic plan, improving quality of life during and after cancer treatment.

This study has some limitations that should be acknowledged. First, additional audiological assessments, such as pure-tone audiometry and speech perception testing, were not performed because participants were evaluated during active chemotherapy in the oncology unit, where their clinical condition often precluded transportation to an audiology clinic and participation in lengthy behavioral assessments. DPOAEs were therefore selected as an objective, rapid, and non-behavioral method suitable for this clinical setting. Second, although treatment information was available in the medical records, cumulative chemotherapy doses, number of treatment cycles, and concomitant use of other potentially ototoxic medications (e.g., antibiotics and diuretics) were not analyzed. Given the large variety of chemotherapy regimens included, incorporating these variables would have substantially fragmented the sample and reduced the statistical power of subgroup analyses. Future studies with larger and more homogeneous patient groups should investigate these factors to better characterize the contribution of individual drugs and cumulative exposure to chemotherapy-induced ototoxicity.

## Conclusion

Chemotherapy with different drugs showed signs of ototoxicity in a large number of patients through changes in OAE results. 79% of patients showed a reduction in response, with an incidence of 81% in the group receiving treatments without platinum-based derivatives, which was similar to the incidence in the platinum-based chemotherapy group (76%). These findings suggest that the ototoxic impact of chemotherapy is broader than what has been traditionally established in literature.

Considering that a significant percentage of chemotherapy patients may experience irreversible hearing changes, we recommend that oncology services implement auditory monitoring before, during, and after chemotherapy, regardless of the treatment protocol used. Appropriate referrals for continuous follow-up should be made as needed.

## Data Availability

the Ungdata surveys that support the findings of this study are available from Universidade Federal de Ciências da Saúde de Porto Alegre (UFCSPA), but restrictions apply to the availability of these data, which were used under licence for the current study and so are not publicly available. The data are, however, available upon request and with the permission of UFCSPA.
